# Prognostic impact of CT severity score in childhood cancer with SARS-CoV-2

**DOI:** 10.1186/s43055-021-00563-5

**Published:** 2021-08-11

**Authors:** Marwa Romeih, Mary Rabea Mahrous, Lobna Shalby, Reham Khedr, Sonya Soliman, Reem Hassan, Mohamed Gamal El-Ansary, Amira Ismail, Ahmed Al Halfway, Abeer Mahmoud, Amal Refeat, Iman Zaki, Mahmoud Hammad

**Affiliations:** 1 Radio-diagnosis Department, Children’s Cancer Hospital, Cairo, Egypt; 2 Radio-diagnosis Department, Faculty of Medicine, Helwan University, Cairo, Egypt; 3 Radio-diagnosis Department, National Heart Institute, Cairo, Egypt; 4 Pediatric oncology department, National cancer institute- Cairo university, and children’s cancer hospital Egypt 57357, Cairo, Egypt; 5 Clinical Pathology Department, National Cancer Institute, Children’s Cancer Hospital, Cairo University, Cairo, Egypt; 6 Clinical pathology department, faculty of medicine Cairo university and children cancer hospital 57357, Cairo, Egypt; 7 Department of Critical Care Medicine, Kasr El-Ainy Cairo University, Cairo, Egypt; 8 Department of Pulmonary medicine, Kasr El-Ainy Cairo University, Cairo, Egypt; 9 Clinical Research Department, Children’s Cancer Hospital, Cairo, Egypt; 10 Radio-diagnosis Department, National Cancer Institute, Cairo University, Cairo, Egypt

**Keywords:** COVID-19, CT severity score, Childhood cancer, SARS-CoV-2

## Abstract

**Background:**

CT chest severity score (CTSS) is a semi-quantitative measure done to correlate the severity of the pulmonary involvement on the CT with the severity of the disease.

The objectives of this study are to describe chest CT criteria and CTSS of the COVID-19 infection in pediatric oncology patients, to find a cut-off value of CTSS that can differentiate mild COVID-19 cases that can be managed at home and moderate to severe cases that need hospital care.

A retrospective cohort study was conducted on 64 pediatric oncology patients with confirmed COVID-19 infection between 1 April and 30 November 2020. They were classified clinically into mild, moderate, and severe groups. CT findings were evaluated for lung involvement and CTSS was calculated and range from 0 (clear lung) to 20 (all lung lobes were affected).

**Results:**

Overall, 89% of patients had hematological malignancies and 92% were under active oncology treatment. The main CT findings were ground-glass opacity (70%) and consolidation patches (62.5%). In total, 85% of patients had bilateral lung involvement, ROC curve  showed that the area under the curve of CTSS for diagnosing severe type was 0.842 (95% CI 0.737–0.948). The CTSS cut-off of 6.5 had 90.9% sensitivity and 69% specificity, with 41.7% positive predictive value (PPV) and 96.9% negative predictive value (NPV). According to the Kaplan–Meier analysis, mortality risk was higher in patients with CT score > 7 than in those with CTSS < 7.

**Conclusion:**

Pediatric oncology patients, especially those with hematological malignancies, are more vulnerable to COVID-19 infection. Chest CT severity score > 6.5 (about 35% lung involvement) can be used as a predictor of the need for hospitalization.

## Background

The coronavirus disease 2019 (COVID-19) pandemic is caused by severe acute respiratory syndrome coronavirus 2 (SARS-CoV-2) [1]. Oncology patients are considered at risk for viral infections due to their immunocompromised state, which may result from long-lasting immunosuppression (steroids, antibodies), or chemo- or radiotherapy [2]. As a consequence, such patients presenting with COVID-19 may have poorer outcomes than others. Patients with hematological malignancies, peri-COVID-19 lymphopenia, or baseline neutropenia were reported to have worse COVID-19 outcomes [3]. In pediatric oncology patients, COVID-19 is also considered a major challenge, as the anti-cancer treatments need to continue with as few modifications as possible to be optimally effective, while infection can postpone elective high-risk therapy to ensure the patient’s safety [4–6]. Therefore, it is essential to ensure early identification of COVID-19 cases that may need clinical care either at a hospital or at home.

Reverse-transcription polymerase chain reaction (RT-PCR) is the gold standard for diagnosing COVID-19, but it has a low sensitivity of ~71%, with a high rate of false-negative results [7, 8]. Currently, chest computed tomography (CT) plays a pivotal role in detecting COVID-19 cases, with high sensitivity of ~94% [7, 9]. The chest CT findings of COVID-19 infection are nonspecific and resemble those seen in other viral pneumonia [10, 11] with an appearance of multifocal ground-glass opacity (GGO) and consolidation, predominantly with a peripheral distribution [10, 12, 13].

Fatal hypoxemia is considered one of the main causes of mortality in COVID-19 infection, which increases as lung involvement increases. Therefore, the degree of pulmonary involvement may be an indicator of outcome [14] and CT findings can be used as an imaging surrogate for disease burden [15]. Chest CT severity score (CTSS) is a semiquantitative measure used to correlate the severity of pulmonary involvement on CT with the severity of the disease [12]. Many studies have used different methods in scoring systems according to the number of lung segments used and the proportion of lung involvement, ranging from 20 to 40 [12, 16, 17]. CTSS of COVID-19 was used as a special imaging tool for assessing the severity of COVID-19 infection in the adult patient [12, 16, 18], a prognostic prediction for the clinical course [15, 17], and a prognostic value in hospitalized patients with COVID-19 [19].

The COVID-19 pandemic has increased the demand among healthcare providers, especially those dealing with cancer patients, to predict the need of patients for hospitalization or admission to an intensive care unit (ICU), rather than being managed at home in isolation.

In this study, we aimed to describe appropriate chest CT criteria and the severity of COVID-19 infection in pediatric oncology patients as a special group, to find a cut-off value of chest CTSS that can differentiate (i) mild cases not requiring oxygen support that can be managed at home and (ii) moderate/severe cases that need oxygen support or ICU admission, allowing appropriate management.

## Methods

### Study design and ethical approval

Ethical approval for this study was obtained from our institutional review board and written informed consents for publication of this work were  provided by the patients’ guardians at the time of admission, as per the hospital’s policy.

### Study population (eligibility criteria)

This study included all pediatric oncology patients with RT-PCR analysis of a nasopharyngeal or oropharyngeal swab positive for SARS-CoV-2 from April 1st , 2020, to November 30th, 2020, at our institution, which is a tertiary referral center for pediatric oncology in Egypt.

All suspected COVID-19 patients were presented to COVID-19 clinics for clinical assessment, then chest CT and PCR were done. All positive cases were hospitalized either in isolation unit or intensive care unit according to their clinical status as regards the hospital policy at that time.

The inclusion criteria were as follows: pediatric oncology patients (≤ 18 years old) with confirmed COVID-19 infection, who underwent an initial chest CT scan at the time of diagnosis. The exclusion criteria were patients with non-COVID-19-related lung pathology (e.g., malignant diseases and/or chronic interstitial disease) or no initial chest CT scan at the time of diagnosis with COVID-19 infection.

### Data collection

All patients’ data from electronic medical records were reviewed, after which the patients were categorized clinically according to the disease severity score at the time of presentation into one of four groups: asymptomatic, mild, moderate, and severe cases [5].

Asymptomatic cases showed no symptoms of COVID-19 at any time of presentation; mild disease was defined as an illness not requiring hospitalization or potentially needing hospitalization for other clinical and laboratory findings; moderate disease was defined as an illness requiring inpatient management for COVID-19-associated symptoms, such as needing oxygen support but without the need for ICU-level care; and severe disease was defined as an illness requiring ICU-level care for COVID-19-related symptoms.

In this study, the following variables were recorded: date of the first positive PCR for SARS-CoV-2 virus, as well as the date of negativity in PCR or death, and disease duration (time between positive and negative PCR results or death). The demographic data of the patients, presenting symptoms assessment (fever, dyspnea, cough, loss of smell, taste, diarrhea, and hypoxia), primary tumor (categorized as hematological or solid), presence or absence of active oncology treatment, and need for O_2_ support and its mode were also recorded. Follow-up of patients was performed for 1 month (once weekly) and chest CT was reviewed in this period to evaluate the disease course.

### CT protocol and imaging data acquisition

Our department specified one CT machine to perform CT exams on COVID-19-infected patients in the scheduled time, aiming to limit the spread of infection to other patients and healthcare workers. In addition, protective infection control measures were implemented.

All patients underwent non-contrast chest CT in a supine position, covering the lung from the apex to the base. A multidetector CT scanner (Somatom Perspective16; Siemens Healthineers, Germany) was used in all cases. The parameters were as follows: tube voltage of 110 kVp, 105 mA, spiral pitch factor of 1.3, the thickness of 1 mm, and increment of 1 mm, with Window baby lung Kernel B60s medium sharp and Window mediastinal Kernel B20s smooth, along with a scan time of 11 s.

### CT image evaluation

Image analysis was performed using the institutional digital database system. Two experienced consultant radiologists (with 10 and 15 years of experience) reviewed the CT images independently at a Philips picture archiving and communication system (PACS) workstation, while blinded to the clinical data. Upon disagreement in the interpretation between the two radiologists, a third experienced radiologist with 25 years of experience adjudicated to give the final decision. The inter-observer agreement was good. No negative control cases were examined.

All of the unenhanced CT images were evaluated in the preset standard pulmonary (width, 1500–2000 HU; level, −450 to 600 HU) and mediastinal (width, 400 HU; level, 60 HU) windows. The following CT characteristics of the lesions were identified according to internationally standard nomenclature [20, 21]. The typical CT findings for COVID-19 included GGO, nodules, consolidation, fibrous bands, pleural effusion, or mediastinal lymphadenopathy defined as lymph node size ≥ 10 mm in short-axis dimension.

The lung abnormalities on CT were evaluated and a semiquantitative scoring system was applied using both axial CT images and multiplanar reconstruction images, based on the scoring system of Li et al. [18] as an easy, uncomplicated, and reproducible system. Each lung lobe (a total of 5 lung lobes; 3 right and 2 left) was scored according to the lung involvement with the following indicators: score 0, 0% involvement; score 1, ≤25%; score 2, 26 to 50%; score 3, 51 to 75%; and score 4, 76% or more. The summation of all five lung lobe scores provided the total CTSS, reflecting the overall lung involvement (the maximum CT score for both lungs was 20) .All follow-up CT scans were performed within 1 month from the date of first positive PCR and the severity scores were calculated by the same method.

### Statistical analysis

Statistical analysis was performed using R for Windows (version 4.0.3). Continuous data not following normal distribution were expressed as sample median with inter-quartile range (IQR). Lung lobe distribution, clinical symptoms, and lung findings of involved lobes were compared in different clinical types by chi-squared test or Fisher’s exact test when the sample size was small. Mann–Whitney test was used for single comparisons, while Kruskal–Wallis test was used for multiple comparisons of CTSS among the different clinical types. A *p* value of less than 0.05 was considered statistically significant. ROC curve was used to test the diagnostic ability of severity score in the mild group and severe group. The Kaplan–Meier method was used to determine the relationship between CTSS and mortality, which was compared using the log-rank test. The intraclass correlation coefficient (ICC) was used to test the consistency of the CTSS of the two observers. ICC values of < 0.4, 0.4–0.75, and > 0.75 represent poor, moderate, and good repeatability, respectively.

## Results

**Fig. 1:**
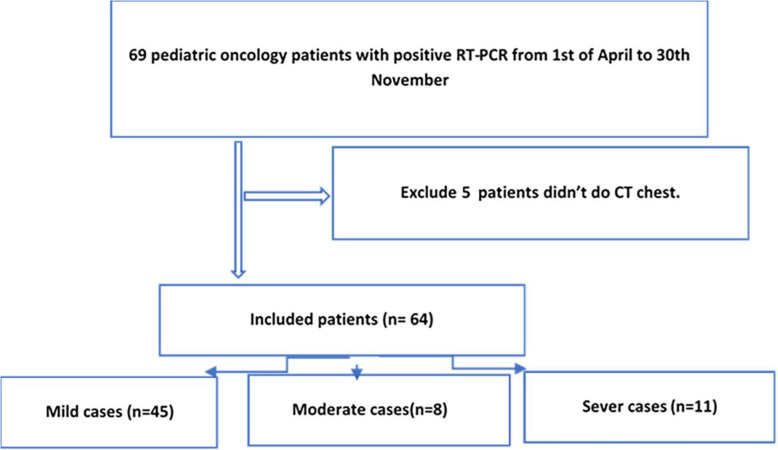
Flow chart

This cohort study of 69 consecutive pediatric oncology patients proven to be positive by RT-PCR for COVID-19 was performed between 1 April and 30 November 2020. Among these 69 patients, we excluded five patients with no available initial chest CT. Therefore, statistical analysis was performed on the 64 patients who met the inclusion criteria for this study (Fig. 1).

### Patient characteristics

The final cohort consisting of 64 pediatric oncology patients with confirmed COVID-19 had a median (IQR) age of 9 [5–15] and 54.7% were male. The age group < 5 years was the least affected (21.87%). Overall, 57 (89%) cases involved hematological malignancies, including 46 (71.8%) patients with leukemia and 7 (11%) with solid tumors. In total, 59 (92%) patients were under active oncology treatment and 5 (8%) were not.

Fever was the most common symptom (92%) in the patients, followed by cough (56.25%), loss of smell and taste (40.62%), along with dyspnea and tachypnea 24 (37.5%). Abdominal symptoms were presented in only 12.5% of the patients (Table 1).

**Table 1:** Summary of patient characteristics

	Characteristic	All patients (***N*** = 64) (%)	Mild (***N*** = 45) (%)	Moderate-severe (***N*** = 19)	***P*** value
**Sex, no. of patients**	Male	35 (54.6%)	26 (57.7%)	9 (47.3%)	0.624^2^
	Female	29 (45.4%)	19 (42.3%)	10 (52.7) %	
**Age range**	< 5 years	14 (21.87%)	11 (24.4%)	3 (15.7%)	0.469^2^
	5-10	27 (42.18%)	20 (44.4%)	7 (36.8%)	
	11-18 years	23 (35.9%)	14 (31.1%)	9 (47.3%)	
**Symptoms**	Fever	59 (92.1%)	41 (91.1%)	18 (94.7%)	1^1^
	Cough	36 (56.25%)	24 (53.3%)	12 (63.15%)	0.654^2^
	Abdominal symptoms	8 (12.5%)	4 (8.8 %)	4 (21%)	0.223^1^
	Dyspnea	24 (37.5%)	5 (11.1%)	19 (100%)	< 0.05^1^
	Loss of smell and taste	26 (40.62%) 16 not identified	22 (48.8%)	4 (21%)	< 0.05^1*^
**Type of primary tumor**	Hematological	54 (84.37%)	42 (93.3 %)	15 (79%)	0.182^1^
	Solid tumor	7 (10.93%)	3 (6.6%)	4 (21%)	

^1^Fisher’s exact test, ^2^chi-squared test

*p < 0.05 defines a significant difference

Mild cases were classified as patients who did not need oxygen support with or without minor CT findings and who could be managed at home under isolation, while moderate and severe cases needed hospital-level care (oxygen support in either the ward or in an ICU; invasive ventilation in severe cases). All of these patients were symptomatic. The mild group represented 70.3% of the total, the moderate group represented 12.5%, and the severe group represented 17.2%. There were thus a relatively small number of cases in both moderate and severe groups, so these were merged into a single moderate/severe group.

Dyspnea and tachypnea mainly occurred in the moderate/severe group (*p* value < 0.05), while the mild group predominantly presented loss of smell and taste (48.8% vs. 21%) (*P* value = 0.0007) (Table 1). There were no significant differences in terms of patient age, sex, and type of primary tumor between the two groups (Table 1).

The median (IQR) disease duration in the mild group was 12 [7–22] days, in surviving moderate/severe patients it was 11 [7–17] days, and in non-surviving severe patients, it was 11 [8–19] days. Severe disease resulted in death in 10 out of 11 patients who needed ventilation. Of these, 9 patients had hematological malignancies and 1 had a solid tumor. The mortality was due to respiratory failure alone or as part of multiorgan failure.

### Chest CT features

All patients underwent initial chest CT along with diagnostic PCR. The chest CT findings varied, including GGO (70%), consolidation patches with air bronchogram (62.5%), pulmonary nodules (36%), and atelectatic fibrous bands (25%) (Table 2). Consolidation patches and the combination of GGO and consolidation patches were significantly more common in the moderate/severe group (*p* = 0.020 and 0.007, respectively) (Table 2). The moderate/severe group was also shown to be significantly more likely to have extrapulmonary findings in the form of pleural effusion (31.5%) and pericardial effusion (21.05%) (*P* <0.015 and *p* = 0.024) (Table 2).

**Table 2:** Chest CT criteria in COVID-19 patients

Findings	Total patient number	Mild (*N* = 45)	Moderate/severe (*N* = 19)	*P* value
Pulmonary findings				
Ground-glass opacity	45 (70.3%)	29 (64.4%)	16 (84.2%)	0.114^2^
Consolidation	40 (62.5%)	24 (53.5%)	16 (84.2%)	0.020^2^*
GGO+consolidation	29 (45.3%)	15 (33.3%)	14 (73.6%)	0.007^2^*
Pulmonary nodules	23 (35.9%)	15 (33.3%)	8 (42.1%)	0.701^2^
Fibrous bands	16 (25%)	8 (17.7%)	8 (42.1%)	0.082^2^
Extrapulmonary findings				
Lymph nodes	9 (14.05%)	4 (8.8%)	5 (26.3%)	0.111^1^
Pleural effusion	9 (14.07%)	3 (6.6%)	6 (31.5%)	0.015^1^*
Pericardial effusion	5 (7.8%)	1 (2.2%)	4 (21.05%)	0.024^1^*

^1^Fisher’s exact test, ^2^chi-squared test

*p < 0.05 defines a significant difference

Bilateral lung involvement was seen in 54 patients (85%). Normal initial chest CT was noted in 4 patients (6%). According to the number of lobes affected, diffuse lung involvement (five lobes were affected) was noted in 25 patients (39%), and no lobes were affected in 4 patients (6.4%).

The right lower lobe showed a greater tendency to be involved (in 85.9% of cases), which was significantly more common in moderate to severe cases (94.8%) than in mild ones (82.3%) (*p* = 0.012) (Table 3).

**Table 3:** The percent of lung lobe involvement (*p < 0.005 defines a significant difference)

	All patients (*N* = 64)	Mild (*N* = 45)	Moderate/severe (*N* = 19)	*P* value
Right upper lobe				0.001*
0	23 (35.9%)	21 (46.68%)	2 (10.5%)	
1	23 (35.9%)	16 (35.5%)	7 (36.8%)	
2	8 (12.5%)	4 (8.8 %)	4 (21%)	
3	2 (3.12%)	1 (2.2%)	1 (5.2%)	
4	8 (12.5%)	3 (6.6 %)	5 (26.3%)	
Right middle lobe				0.001*
0	34 (53.1%)	29 (64.4%)	5 (26.3%)	
1	15 (23.4%)	10 (22.2%)	5 (26.3%)	
2	8 (12.5%)	4 (8.8 %)	4 (21%)	
3	3 (4.7%)	1 (2.2%)	2 (10.5%)	
4	4 (6.3%)	1 (2.2%)	3 (15.7%)	
Left upper lobe				0.002*
0	28 (43.75%)	24 (53.3%)	4 (21%)	
1	18 (28.12%)	13 (28.8%)	5 (26.3%)	
2	8 (12.5%)	4 (8.8%)	4 (21%)	
3	5 (7.81%)	3 (6.6%)	2 (10.5%)	
4	5 (7.81%)	1 (2.2%)	4 (21%)	
Right lower lobe				0.012*
0	9 (14.1%)	8 (17.7%)	1 (5.2%)	
1	20 (31.2%)	15 (33.3%)	5 (26.3%)	
2	15 (23.4%)	13 (28.8%)	2 (10.5%)	
3	12 (18.8%)	6 (13.3)	6 (31.5%)	
4	5 (7.81%)	5 (26.3%)	5 (26.3%)	
Left lower lobe				0.006*
0	11 (17.2%)	9 (20%)	2 (10.5%)	
1	23 (35.9%)	19 (42.2%)	4 (21%)	
2	13 (20.3%)	10 (22.2%)	3 (15.7%)	
3	9 (14.1%)	5 (11.1%)	4 (21%)	
4	8 (12.5%)	2 (4.4) %	6 (31.5%)	

A higher CTSS was noted in both the right upper lung lobe and the left lower lung lobe about 12.5% in the study population. The most preserved (least affected) lobes were the right middle lobe (about 53.1% of our population showed a score of 0) and the left upper lobe (43.75%).

Inter-observer test results of CT visual quantitative analysis of the two observers showed good repeatability, with ICC of 0.976 (95% confidence interval 0.962–0.985).

### The relationship between CT severity score and clinical severity

In severe cases, the initial CTSS median (IQR) was 9 (7–12.5), while in moderate cases it was 8.5 (4.5–19.75), and that in mild cases was 5 [3–7]. CTSS was compared among the three groups (mild/moderate/severe). A statistically significant difference was found when all groups were compared together (*p* = 0.001).

**Fig. 2:**
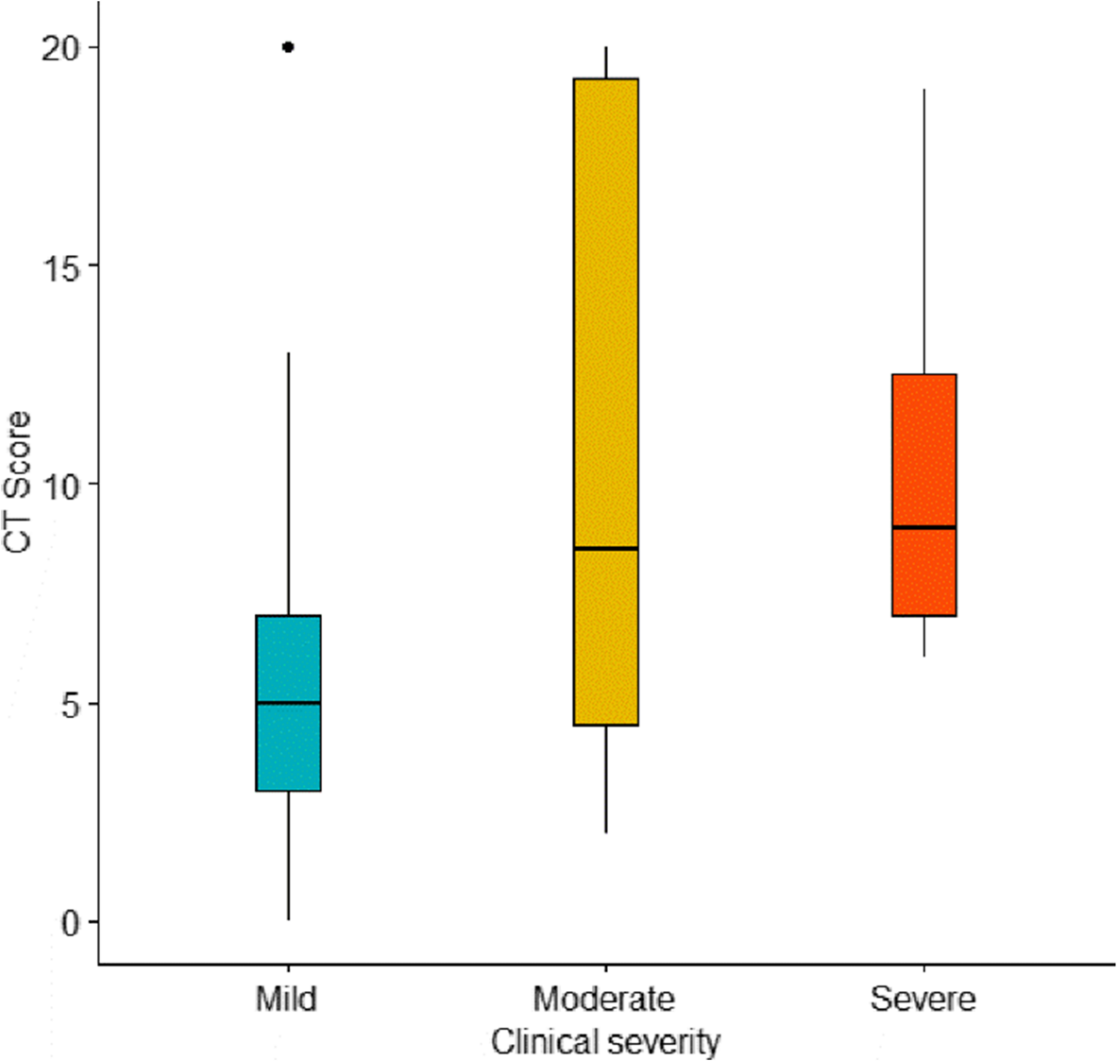
CT severity score compared among the three groups (mild/moderate/severe)

When multiple comparisons were performed, the CTSS was significantly higher in the severe group than in the mild group (*p* = 0.001), while no statistically significant difference was found between the mild and moderate groups (*p* = 0.094) or between the moderate and severe groups (*p* = 0.740) (Fig. 2).

**Fig. 3:**
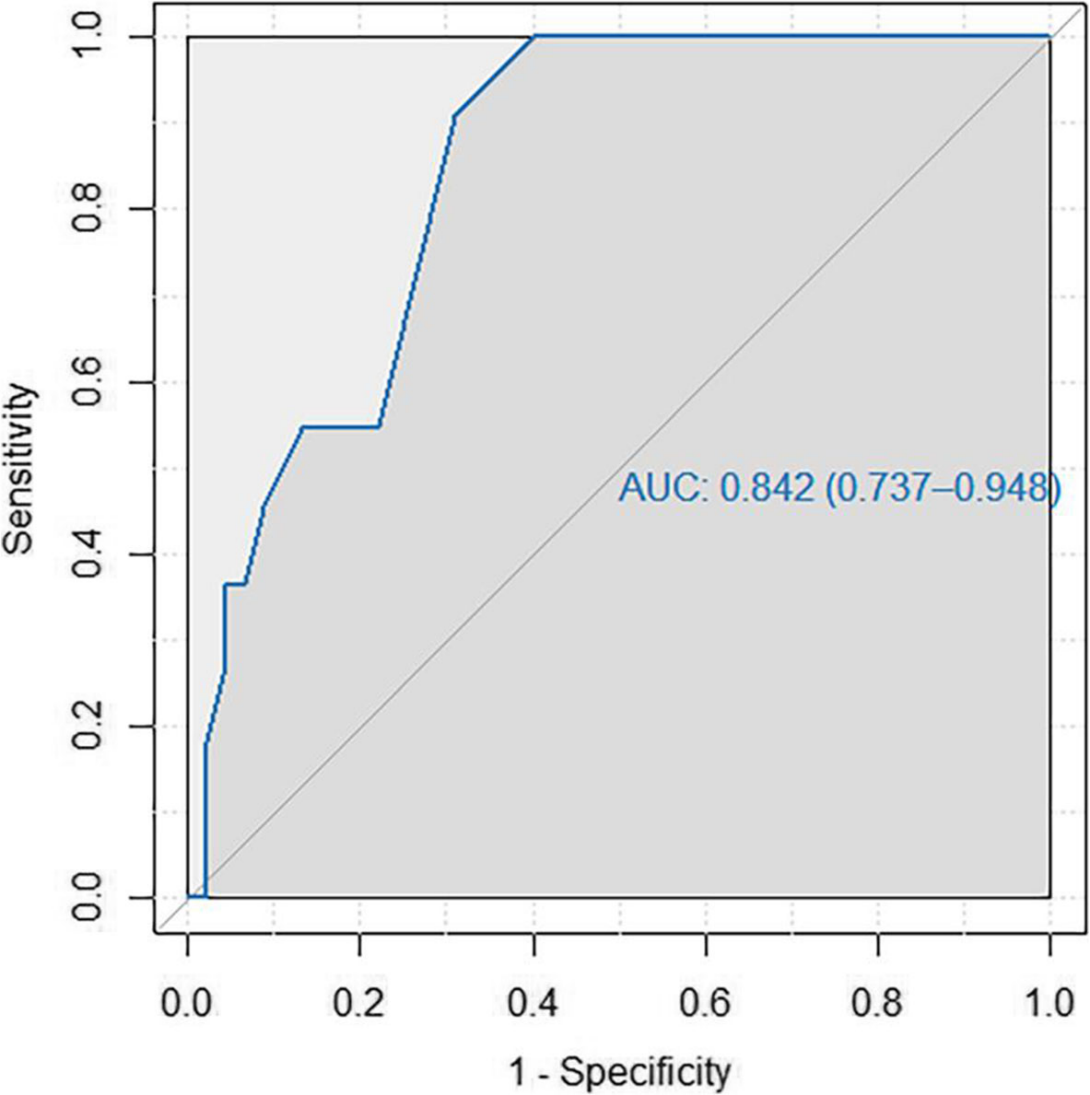
ROC curve to test the cut-off point of CT severity score between the mild group and severe group

ROC curve was used to test the ability of CTSS to differentiate between the mild group and the severe group. ROC curve  showed that the area under the curve (AUC) of CTSS for diagnosing severe type was 0.842 (95% CI 0.737–0.948). The CTSS score cut-off of 6.5 had 90.9% sensitivity and 69% specificity (Fig. 3).

The numbers of patients with a CTSS greater than 6.5 were 10 in the severe group and 14 in the mild group. The corresponding numbers of patients with a CTSS of less than 6.5 were 1 and 31, respectively, resulting in a positive predictive value of 41.7% and a negative predictive value of 96.9%.

Applying this cut-off value to the moderate/severe group, we found that the numbers of patients with a CTSS greater than 6.5 were 14 in the moderate/severe group and 14 in the mild group. Moreover, the corresponding numbers of patients with a CTSS less than 6.5 were 5 and 31, respectively, resulting in a positive predictive value of 50% and a negative predictive value of 86.1%.

In the moderate/severe group, four out of five patients who had a CTSS of less than 6.5 showed pericardial effusion, while most of the 14 mild group patients presenting with a CTSS of more than 6.5 were neutropenic and had superadded bacterial infection [methicillin-resistant *Staphylococcus aureus*, Staphylococcus, etc.] and improved with appropriate antibiotics. There were no statistically significant differences in CTSS between the age groups or sexes (*p* = 0.627 and *p* = 0.90, respectively).

The patients were followed up for 1 month (chest CT was performed weekly). Eleven cases were excluded from follow-up evaluation: 7 in the moderate/severe group as they died early in the course of the disease and 4 in the mild group who had an initially normal chest CT.

**Fig. 4:**
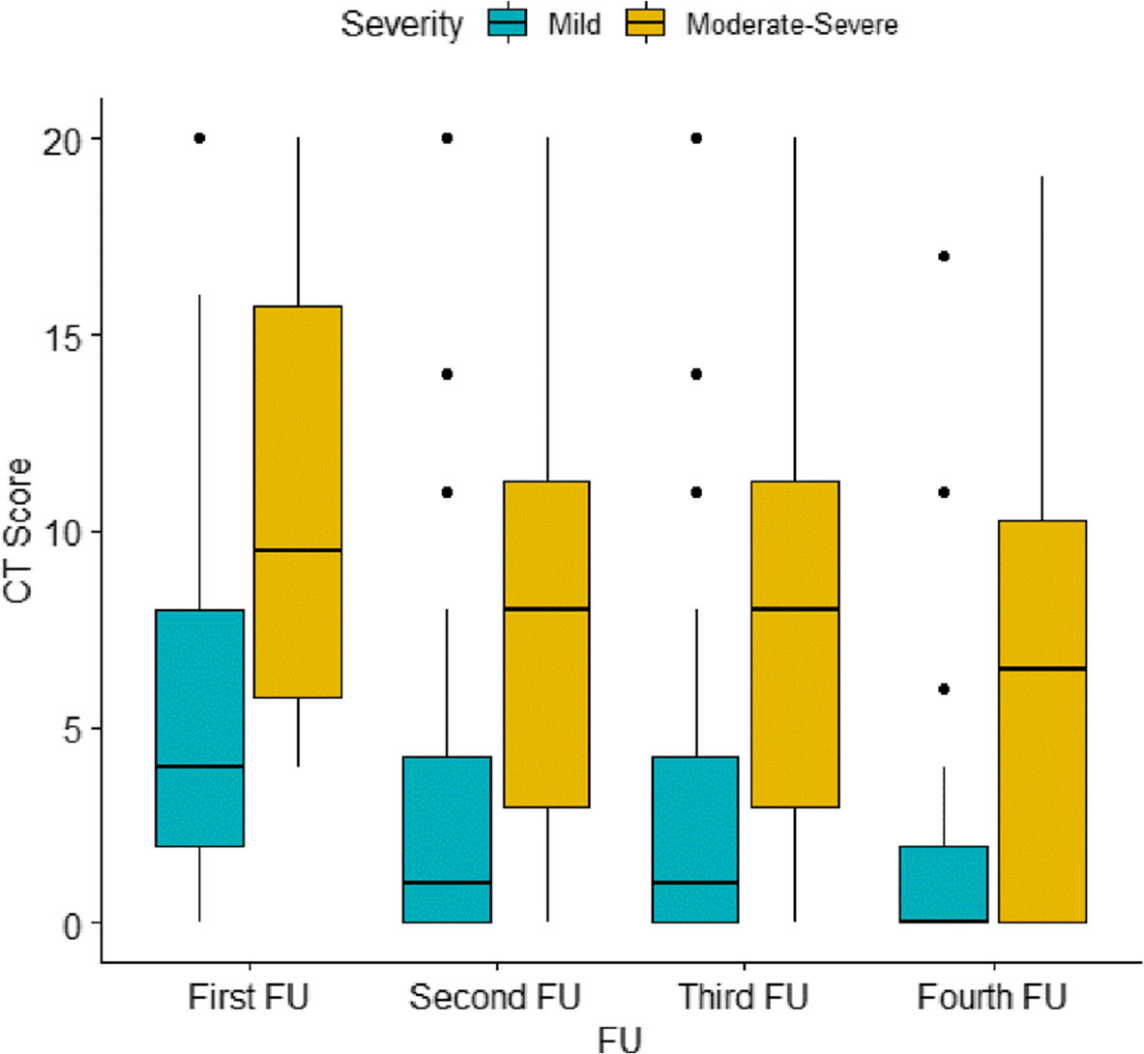
The median chest CT score was plotted against the 1-month follow-up period for mild and moderate to severe COVID-19 patients

The median (IQR) of the 1st week follow-up CTSS in the mild group was 4 [2–8] and that in the moderate/severe group was 9.5 (5.75–15.75). However, in the 4th week of follow-up, the median of the mild group was 0 (0–2) (normal chest CT), and that in the moderate/severe group was 6.5 (0–10.25) (Fig. 4).

### Survival analysis

**Fig. 5:**
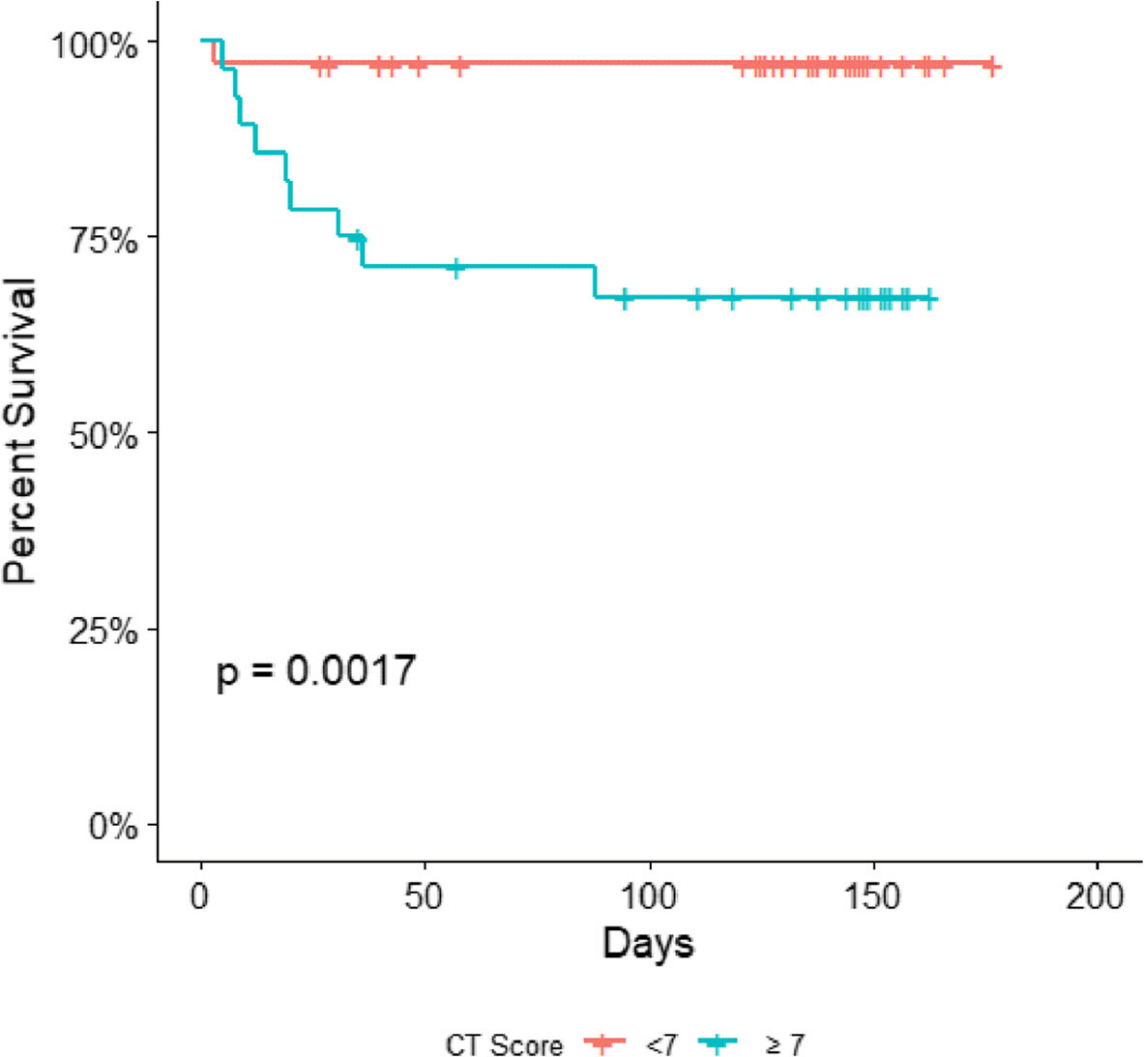
Kaplan–Meier survival curve. Estimated survival rate comparison between COVID-19 patients with CT scores of < 7 and ≥ 7. Percentage survival is expressed on the *y*-axis, while time (days) of the observation period is expressed on the *x*-axis

Kaplan–Meier survival curves were used to compare the survival rates between COVID-19 patients with CTSS of < 6.5 and ≥ 6.5 (Fig. 5). According to the Kaplan–Meier analysis, the risk of mortality was higher in patients with a CTSS > 7 than in those with a CTSS < 7 (log-rank, *p* = 0.0017, hazard ratio = 12.8, CI = 1.6–101%).

## Discussion

Few studies have dealt with pediatric oncology patients infected with COVID-19 [4–6, 22]. In addition, to the best of our knowledge, no studies have evaluated the prognostic value of chest CT in COVID-19-infected pediatric oncology patients. This study involved a semiquantitative assessment of CTSS in pediatric oncology patients with COVID-19, concerning the clinical presentation, need for oxygen support, and outcomes.

According to CTSS, as it was used to differentiate between the mild and moderate/severe groups. The ROC analysis showed that the AUC was 0.842 (95% CI 0.737–0.948) and the cut-off of 6.5/20 (with about 32.5% lung involvement) had 90.9% sensitivity and 69% specificity. This was close to the 9/25 (about 36% lung involvement) cut-off for CTSS used for hospital admission in a multi-center prospective study in adults, as reported by Awe et al. [23], with a sensitivity of 65.4% and specificity of 78.1%. It is also in agreement with the findings of Shi et al. [24] for a CTSS cut-off for identifying severe-critical type in adults of 7.5/20 (37.5%), having 82.6% sensitivity and 100% specificity. Meanwhile, Yang et al. [12] determined a CT cut-off score of 19.5 out of 40 (48.7%) for identifying severe patients, with 83.3% sensitivity and 94% specificity. The difference in the latter may be explained by the different categories of adults in this study.

In the current study, no statistically significant difference was found between CT severity score and different age categories, in contrast to the finding of Steinberger et al. [25] that there is a correlation between increasing age and an increasing CTSS. This difference may be explained by the presence of a common association (malignancy and immunosuppressed state) in our group of patients.

Overall survival analysis was performed to compare the survival rates between COVID-19 patients with CTSS of < 7 and ≥ 7, to confirm the prognostic impact of chest CT findings over the 1-month follow-up period. We demonstrated that a cut-off value of 7 had a high predictive value for mortality in the first month. Francone M [17] do a survival analysis over an observational period of 24 days on 1274 adult patients with COVID-19 infection and determined a cut-off value of ≥ 18/25 CTSS is highly predictive of short-term mortality.

**Fig. 6:**
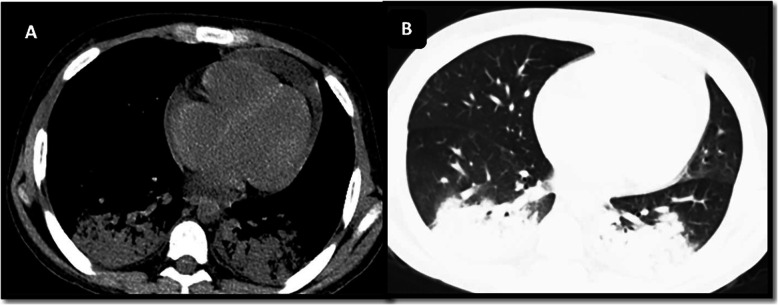
A male patient aged 10 years old with ALL and under chemotherapy developed COVID-19 infection presenting with dyspnea, tachypnea, and fever (moderate clinical presentation). Chest CT revealed pericardial effusion and bilateral basal consolidative patches, with a severity score of 4 (**A**, mediastinal window; **B**, lung window)

The moderate to severe group was more likely to have extrapulmonary findings in the form of pleural effusion (31.5%) and pericardial effusion (21.05%) (Fig. 6), with statistically significant differences (*p* < 0.015 and *p* = 0.024), in agreement with the work of Li et al. [26] who found that pericardial effusion could be an indicator of severity. This may be explained by associations with pleuritis, pericarditis, or myocarditis, as many recent studies proved that COVID-19 may present as a multisystem inflammatory syndrome.

Regarding the patient characteristics and clinical presentation, the age group < 5 years was the least affected group (21.87%), followed by the group aged 11–18 years (35.9%). This is in agreement with the work of Madhusoodhan et al. [27] but in contrast to that of Roganovic [6], who reported that children aged ≤ 5 years were more vulnerable to infection than older children. This may be attributed to the lower ACE2 gene expression in children’s nasal epithelium [22, 28] and those older children (11–18 years) are used to complying with hygiene rules in the form of frequent handwashing and mask-wearing, and to avoid circumstances associated with a risk of infection.

In agreement with our results, the predominance of infection among male patients was noted in the literature on pediatric as well as adult populations [4–6, 15, 25, 29]. Patients with hematological malignancies were the group most infected with COVID-19 (89%), and 71.8% of them had leukemia, while about 92% of patients were receiving chemotherapy. This is in agreement with recent studies documenting that patients with active hematological malignancies were at higher risk of COVID-19 [3–6, 30].

The majority of cases were mild (70.3%), which is in agreement with the work of Madhusoodhan et al. [27] (61.6%) and Montoya et al. [4] in the field of pediatric oncology. Moreover, several prior studies reported that COVID-19 is generally milder in children than in adults [17, 31, 32].

Fever was the most common presenting symptom at the onset of illness (92.1%), followed by cough (56.25%), in agreement with the findings of Roganovic [6], Montoya et al. [4], and Boulad et al. [5] in pediatric oncology patients as well as Dong et al. [28], Hrusak et al. [33], and Xia et al. [32] in studies on immunocompetent pediatric patients. Dyspnea and tachypnea were mainly found in the moderate/severe group (*p* value < 0.05), in agreement with the findings of Chen et al. [34] and Allali et al. [35] who found that higher mortalities were linked to patients presenting with dyspnea.

**Fig. 7:**
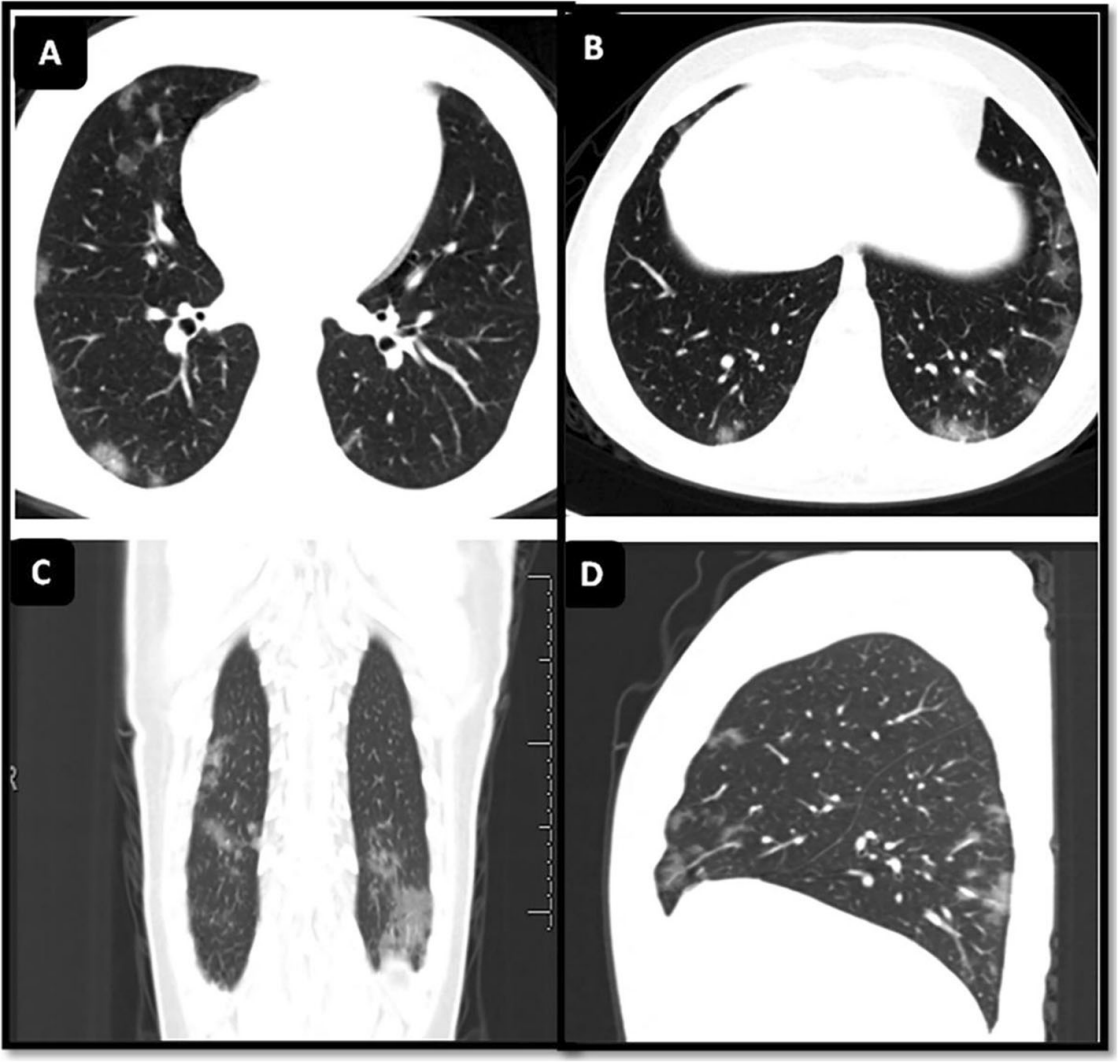
A male patient aged 8 years old with AML and under chemotherapy developed a COVID-19 infection and presented with fever and cough. Chest CT revealed multiple bilateral peripherally located ground-glass opacities; chest CT severity score was 5

Regarding the chest CT pattern and distribution, the rate of negative CT findings in our study was 6%, which was significantly lower than those in studies by Steinberger et al. [25] and Chen et al. [36] who studied pediatric immunocompetent populations with no comorbidities. The most common findings were bilateral GGO (70%) (Fig. 7) and consolidation (62.5%), and a mixed pattern of both GGO and consolidation (45.3%), consistent with data reported in previous studies on either an adult or a pediatric population [24, 36–39].

Consolidation patches and a mixed pattern of GGO and consolidation were significantly more prevalent in the moderate to severe group than in the mild group (*p* = 0.02 and 0.00719, respectively), in agreement with the findings of Li et al. [40] and Yuan et al. [41] who reported the presence of consolidation in severe and high-mortality groups. This may be explained by consolidation usually being associated with a progressive form of the disease, which causes more damage to the alveolar wall [36].

Bilateral lung involvement was seen in 85% of patients, which is more frequent than bilateral involvement in the pediatric immunocompetent population with no comorbidities (71%) as presented by Steinberger et al. [25], but it was closer to that noted in adult populations (76–82%) [11, 42].

The right lower lobe showed a greater tendency to be involved (in 85.9% of cases), compared with 93.8% in the study by Francone et al. [17], which was significantly more commonly affected in moderate to severe cases (94.8%) than in mild ones (82.3%) (*p* = 0.012). Left lower lung lobe (seen affected in 82.8% of cases) compared to 94.6% in Francone et al. [17] with more affection in moderate to severe cases (89.5%) compared to 82.8% in mild cases showing statistical significance difference (*p* = 0.006).

**Fig. 8:**
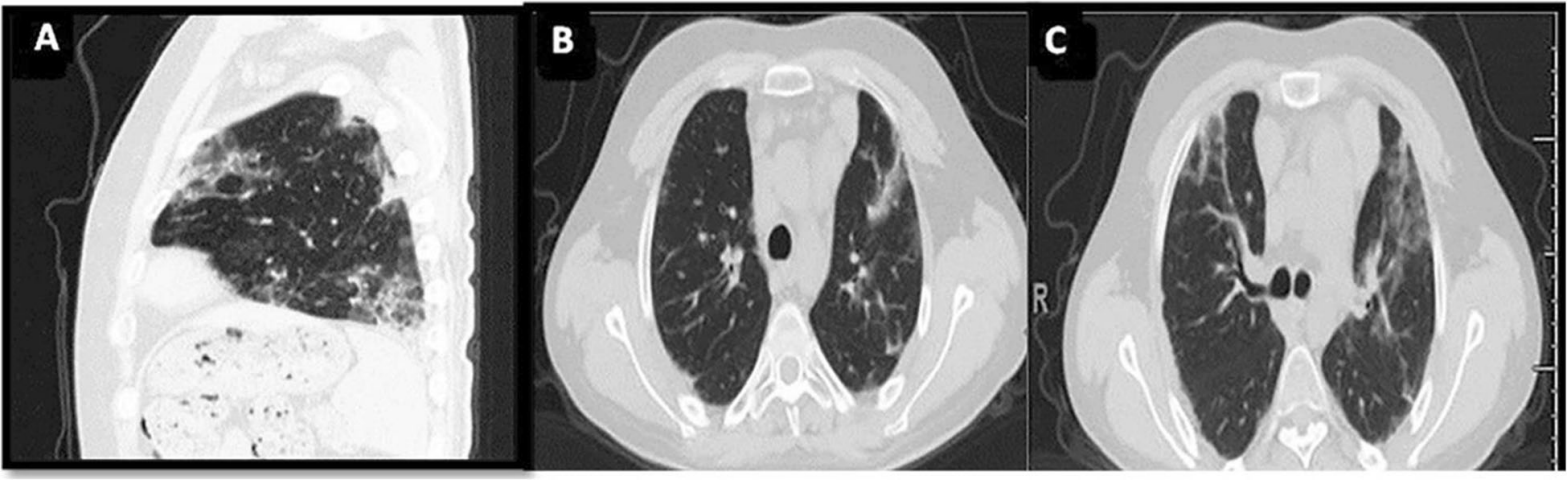
Fourth-week follow-up chest CT of a female patient aged 12 years old with ALL and receiving chemotherapy, who developed COVID-19 infection presenting with dyspnea, tachypnea, and fever. The patient needed O_2_ at the time of presentation. The initial chest CT severity score was 18 and, 1 month later, there were residual consolidation patches, linear opacities, crazy-paving patterns, and linear opacities. Chest CT severity score was 8

Chest CT of almost all mild cases became clear within 1 month, but moderate/severe cases took longer to recover. The most common findings were residual consolidation patches, linear opacities, crazy-paving patterns, and linear opacities (Fig. 8).

There are many limitations in the present study. First, there was no comparative group including normal immunocompetent pediatric patients, so the generalizability on the pediatric group was not being allowed. Second, this study had a retrospective, single-center design with relatively small sample size and incomplete CT follow-up in severe patients, as chest CT was not easy to perform in severely distressed patients in the ICU. According to our knowledge, this first study found a cut-off value for CT chest severity score in pediatric oncology patients as a prognostic factor so we recommend future larger/multi-center studies to validate this value and better clarify its impact on clinical decision-making. Finally, many factors might contribute to the disease outcome, such as the stage of primary disease, the general condition of the patient, and the type of chemotherapy.

## Conclusion

Pediatric oncology patients, especially those with hematological malignancy, are more vulnerable to COVID-19 infection and some can exhibit an aggressive course. Chest CT besides PCR can be a definitive diagnostic method for SARS-COV-2 infection. Furthermore, a chest CT severity score of > 6.5 (about 35% lung involvement) can be used as a predictor of disease severity and early need for hospitalization. Chest CT is recommended as a rapid triage tool and sensitive gatekeeper to categorize pediatric oncology patients with COVID-19 who need to be hospitalized.

## Data Availability

The datasets used and analyzed during the current study are available from the corresponding author on reasonable request.

## Abbreviations

COVID-19: Coronavirus disease 2019
SARS-CoV-2: Severe acute respiratory syndrome coronavirus 2
RT-PCR: Reverse-transcription polymerase chain reaction
CT: Computed tomography
GGO: Ground-glass opacity
CTSS: Chest CT severity score
ICU: Intensive care unit
PACS: Picture archiving and communication system
HU: Hounsfield units
IQR: Inter-quartile range
ROC: Receiver operating characteristic
ICC: Intraclass correlation coefficient
AUC: The area under the curve
ACE2: Angiotensin-converting enzyme
